# Unravelling Human Trypanotolerance: IL8 is Associated with Infection Control whereas IL10 and TNFα Are Associated with Subsequent Disease Development

**DOI:** 10.1371/journal.ppat.1004469

**Published:** 2014-11-06

**Authors:** Hamidou Ilboudo, Rachel Bras-Gonçalves, Mamadou Camara, Laurence Flori, Oumou Camara, Hassane Sakande, Mamadou Leno, Elodie Petitdidier, Vincent Jamonneau, Bruno Bucheton

**Affiliations:** 1 Centre International de Recherche-Développement sur l'Elevage en zones Subhumides (CIRDES), Unité de Recherches sur les Bases Biologiques de la Lutte Intégrée, Bobo-Dioulasso, Burkina Faso; 2 Institut de Recherche pour le Développement (IRD), UMR IRD-CIRAD 177 INTERTRYP, Campus International de Baillarguet, Montpellier, France; 3 Ministère de la Santé et de l'Hygiène Publique, Programme National de Lutte contre la Trypanosomose Humaine Africaine, Conakry, Guinée; 4 Centre de coopération Internationale en Recherche Agronomique pour le développement (CIRAD), UMR IRD-CIRAD 177 INTERTRYP, Campus International de Baillarguet, Montpellier, France; 5 Institut National de la Recherche Agronomique (INRA), UMR 1313 GABI, F78350 Jouy-en-Josas, France; Institute of Tropical Medicine, Belgium

## Abstract

In West Africa, *Trypanosoma brucei gambiense*, causing human African trypanosomiasis (HAT), is associated with a great diversity of infection outcomes. In addition to patients who can be diagnosed in the early hemolymphatic phase (stage 1) or meningoencephalitic phase (stage 2), a number of individuals can mount long-lasting specific serological responses while the results of microscopic investigations are negative (SERO TL+). Evidence is now increasing to indicate that these are asymptomatic subjects with low-grade parasitemia. The goal of our study was to investigate the type of immune response occurring in these “trypanotolerant” subjects. Cytokines levels were measured in healthy endemic controls (n = 40), stage 1 (n = 10), early stage 2 (n = 19), and late stage 2 patients (n = 23) and in a cohort of SERO TL+ individuals (n = 60) who were followed up for two years to assess the evolution of their parasitological and serological status. In contrast to HAT patients which T-cell responses appeared to be activated with increased levels of IL2, IL4, and IL10, SERO TL+ exhibited high levels of proinflammatory cytokines (IL6, IL8 and TNFα) and an almost absence of IL12p70. In SERO TL+, high levels of IL10 and low levels of TNFα were associated with an increased risk of developing HAT whereas high levels of IL8 predicted that serology would become negative. Further studies using high throughput technologies, hopefully will provide a more detailed view of the critical molecules or pathways underlying the trypanotolerant phenotype.

## Author Summary

Whereas immunological mechanisms involved in the control of trypanosome infections have been extensively studied in animal models, knowledge of how *Trypanosoma brucei gambiense* interacts with its human hosts lags far behind. In this study we measured cytokine levels in sleeping sickness patients and individuals who were apparently able to control infection to subdetection levels over long periods of time or who were engaged in a process of self-cure as demonstrated by the disappearance of specific antibodies. In contrast to patients, trypanotolerant subjects were characterized by a strong inflammatory response with elevated levels of IL8, IL6, and TNFα. This study indicates that both protective immune responses and markers of disease development exist in human *T. brucei. gambiense* infection and constitute an important step forward to identify new diagnostic or therapeutic targets in the fight against sleeping sickness.

## Introduction

Human African trypanosomiasis (HAT), or sleeping sickness, caused by *Trypanosoma brucei gambiense* (*T. b. gambiense*) is classically described as a chronic infection characterized by an early hemolymphatic stage (stage 1) associated with nonspecific symptoms such as intermittent fevers and headaches, followed by a meningoencephalitic stage (stage 2) in which the parasite invades the central nervous system and causes neurological disorders and death if left untreated. Long considered as invariably fatal, observations are increasingly indicating that infection by *T. b. gambiense* can result in a wide range of clinical outcomes in its human host [1]–[3]. Recently, self-cure processes have been described in HAT patients refusing treatment in Côte d'Ivoire [4]. Furthermore, individuals with (i) high responses to the card agglutination test for trypanosomiasis (CATT); (ii) serological positivity to the highly specific *T. b. gambiense* immune trypanolysis test (TL) and (iii) negative parasitological results (SERO TL+) have been reported from a number of endemic foci in West Africa [5], [6]. Noteworthy, follow-up studies showed that only some of these subjects develop HAT (parasite can be detected by microscopy in body fluids) while others are able to maintain high and specific serological responses over long periods of time [7]. These observations suggest that SERO TL+ individuals have been in contact with *T.b. gambiense* and that some of them are able to control infection to levels that cannot be detected by microscopy. This hypothesis is supported by the fact that parasite DNA can be detected by PCR in this category of subjects [7], [8] and that direct microsatellite typing of trypanosomes from blood samples detected the same genotypes as those found in HAT patients [9]. Overall, these field observations are in line with the idea that trypanotolerance exists in humans, too, as demonstrated in some West African taurine breeds and in inbred mice models displaying differential susceptibility toinfection [10], [11].

While several studies were designed to investigate the immune response in HAT patients at different stages of disease [12], [13], almost nothing is known about the response occurring in SERO TL+ subjects who are apparently able to control infection. In this study, we evaluated the levels of 10 cytokines (IL12p70, IL2, IL4, IL5, IL8, IL1β, IL6, IL10, tumor necrosis factor (TNF)α, and interferon (INF)γ) in HAT patients, SERO TL+ subjects and endemic controls recruited during medical surveys which were conducted in active HAT foci in Guinea. In addition, SERO TL+ subjects were followed up for at least 2 years in order to analyze the prognostic value of cytokine levels determined at study inclusion on the subsequent evolution of the serological and parasitological status: (i) development of HAT; (ii) maintenance of high antibody responses, and (iii) progressive decrease in antibody responses.

## Materials and Methods

### Study population and definition of phenotypes

The study was carried out in three active HAT foci (Dubreka, Boffa, and Forecariah) located in mangrove areas of coastal Guinea [14]. Most of the population is from the Soussou ethnic group and lives in small villages scattered along mangrove channels. Main occupations are rice cultivation, fishing, wood cutting, and salt extracting, all activities that bring the population into close contact with *Glossina palpalis gambiensis* which is the only vector of *T. b. gambiense* in these areas [15], [16]. Other diseases such as tuberculosis, leprosy or cholera are still present and malaria is highly endemic.

All subjects included in this study were identified during medical surveys organized by the National Control Programme (NCP) between November 2007 and May 2011, according to the WHO and NCP policies, as described previously [7]. During the surveys a total of 41,311 individuals were screened, blood (5 ml) was collected in heparanized tubes from all individuals who tested positive to the CATT mass screening test, and a twofold plasma dilution series was tested to determine the CATT end titer. All individuals with end titers of 1/8 or greater were submitted to microscopic examination of lymph node aspirates whenever swollen lymph nodes were present; 350 µl of buffy coat was then examined by using the mini-anion exchange centrifugation test which has shown to have a positive threshold of 10 trypanosomes/ml of blood [15]. When trypanosomes were detected, lumbar puncture was performed and the disease stage determined by searching for trypanosomes using the modified simple centrifugation technique [17] of cerebrospinal fluid (CSF) and by white blood cell (WBC) counts. HAT patients were classified as stage 1 (0–5 WBC/µl), early stage 2 (6–20 WBC/µl; or ≤20 WBC with trypanosomes in CSF), or late stage 2 (>20 WBC/µl) and treated according to the NCP recommendations. In addition to HAT patients (n = 108) and CATT plasma-positive subjects (SERO, n = 84), 5 ml of blood was also taken from CATT-negative individuals (n = 42) selected from the same CATT series as the HAT and SERO subjects and subjected to the same tests as described above. For all subjects, aliquots were made with leftover plasma and with CSF for HAT patients when available. Collected samples were then frozen directly in the field in a liquid nitrogen container and stored at −80°C at CIRDES until use. For each individual, an aliquot of plasma was used to perform the immune trypanolysis test that detects Litat 1.3 and Litat 1.5 variable surface antigens specific for *T. b. gambiense* [6]. The 24 SERO that were negative and the two endemic controls that were positive were excluded at this stage of the study; all HAT patients who were positive and those for whom both plasma and CSF samples were available (n = 52) were included in the study: 10, 19, and 23 were classified as stage 1, early stage 2, and late stage 2, respectively. The study sample phenotypic and demographic characteristics are summarized in Table 1. All SERO TL+ individuals were then followed up at their home for 2 years. When present, serological and parasitological tests were repeated as described above. Out of the 60 SERO TL+, 40 could be followed up for at least 2 years (on average 3 visits) and were included in the analysis of the prognostic value of cytokine levels in the evolution of the serological and parasitological status.

**Table 1:** Phenotypic and demographic characteristics of the study sample.

Phenotypic groups	Inclusion criteria's	N	mean age [range]	ratio male/female
**Endemic controls**	CATT negative	40	39.4 [17–80]	1.5
	trypanolysis test negative			
	no parasite detected in body fluids			
**HAT patients**	CATT≥1/8	52	29.4 [4–70]	1.5
	trypanolysis test positive			
	parasites detected in body fluids			
**SERO TL+**	CATT≥1/8	60	33.1 [5–70]	1.1
	trypanolysis test positive			
	no parasite detected in body fluids			

### Cytokines assays

Plasma cytokine levels (IL12p70, IL2, IL4, IL5, TNFα, INFγ, IL8, IL1β, IL6, and IL10) were determined for all study subjects with the human Th1/Th2 and human inflammation Cytometry Bead Array (CBA) cytokine kits according to the manufacturer's instructions (BD, Biosciences). For CSF samples we used only the human inflammation CBA kit to quantify IL8, IL1β, IL6, IL10, IL12, and TNFα levels. After acquiring sample data by flow cytometry (BD FACSCanto) and the BD FACSDiva software, results were generated in a graphical and tabular format using the Flow Cytometric Analysis Program Array software (FCAP Array, BD Biosciences).

### Statistical analysis

Univariate analysis of cytokine levels between groups was performed by using the nonparametric Wilcoxon signed-rank test (Kruskal-Wallis). The association between cytokine levels and the risk to develop HAT in SERO TL+ individuals was also evaluated by stepwise multivariate logistic regression. The covariates included in these analyses were age (in years), gender, and cytokine levels. Cytokine levels were assigned to two classes of equal size using the median cytokine value as the threshold. The most significant covariates (P<0.1) were then entered one by one until no significant improvement in the model likelihood ratio was observed. The JMP5 (SAS Institute) software was used for univariate analyses and multivariate logistic regressions and the R software was used for the construction of box-plots. Normed principal component analysis (PCA) was performed using the ade4 package in the R environment [18] with cytokines data ln(1+x) transformed. The association between each of the study recorded covariates and the individuals x and y coordinates on the first factorial plan was assessed by logistic regression for qualitative covariates (HAT status, disease stage in HAT patients, gender and disease geographic focus) and by linear regression for age.

### Ethical considerations

This study was performed as part of medical surveys conducted by the NCP according to the national HAT diagnostic procedures and was approved by the Ministry of Health in Guinea. All participants were informed of the objectives of the study in their own language and signed a written informed consent form. For participants under 18 years of age, a written informed consent was obtained from the parents. This study is part of a larger project aiming to improve HAT diagnosis for which approval was obtained from the World Health Organization (WHO, Research Ethics Review Committee) and IRD (Comité Consultatif de Déontologie et d'Ethique) ethical committees.

## Results

### Cytokine levels in CSF and plasma according to disease stage

Mean cytokines levels measured in the CSF and plasma samples of HAT patients in the different disease stages are shown in Table 2. In the CSF, TNFα, IL1β and IL12 levels were similar in all stages. Patients in the second stage of disease had significantly higher IL10 (p = 0.0003), IL8 (p = 0.001), and IL6 (p = 0.01) levels in CSF than patients in stage 1 or early stage 2. In contrast, none of the cytokines measured in the plasma displayed a significant association according to the disease stage although a trend was observed for IL1β and INFγ levels to be increased along with disease severity (Figure S1). On the basis of these results, we gathered all patients into one single group (HAT patients group) in order to further compare the plasma levels of cytokines with those of SERO TL+ and endemic controls.

**Table 2:** Cytokine levels measured in the CSF and plasma of HAT patients according to the disease stage.

		CSF		Plasma	
Cytokines	Stage	median (pg/ml) [IQR]^1^	p-value^2^	median (pg/ml) [IQR]	p-value
	stage 1	29.8 [18.6–50.3]		0.0 [0.0–5.2]	
**IL8**	early stage 2	49.9 [26.3–71.7]		0.0 [0.0–8.8]	
	late stage 2	81.5 [39.7–221.8]	**0.0015**	8.4 [0.0–14.3]	0.1290
	stage 1	0.0 [0.0–5.4]		1.4 [0.0–3.5]	
**IL6**	early stage 2	0.0 [0.0–4.6]		0.0 [0.0–4.8]	
	late stage 2	5.5 [0.8–12.4]	**0.0126**	2.7 [0.0–4.4]	0.7642
	stage 1	0.0 [0.0–3.5]		7.6 [2.1–17.7]	
**IL10**	early stage 2	3.9 [0.0–5.6]		7.9 [4.9–13.6]	
	late stage 2	15.3 [4.5–24.4]	**0.0003**	11.1 [7.1–25.7]	0.4368
	stage 1	0.0 [0.0–0.0]		0.0 [0.0–0.0]	
**TNFα**	early stage 2	0.0 [0.0–4.0]		0.0 [0.0–5.2]	
	late stage 2	0.0 [0.0–6.8]	0.3142	0.0 [0.0–0.0]	0.1719
	stage 1	0.0 [0.0–2.8]		1.2 [0.0–3.3]	
**IL12p70**	early stage 2	2.1 [0.0–2.4]		3.0 [2.2–4.3]	
	late stage 2	0.0 [0–2.6]	0.7009	3.4 [0.0–5.8]	0.3039
	stage 1	0.0 [0.0–0.0]		0.0 [0.0–12.8]	
**IL1β**	early stage 2	0.0 [0.0–0.0]		15.4 [0.0–28.5]	
	late stage 2	0.0 [0.0–0.0]	0.2276	16.5 [0.0–60.1]	0.1673
	stage 1			0.0 [0.0–0.0]	
**IL5**	early stage 2			0.0 [0.0–0.0]	
	late stage 2			0.0 [0.0–0.0]	0.6065
	stage 1			5.7 [0.0–14.8]	
**IL2**	early stage 2			6.4 [4.5–13.1]	
	late stage 2			6.5 [3.7–13.7]	0.5226
	stage 1			4.9 [0.0–8.3]	
**IL4**	early stage 2			3.2 [0.0–10.8]	
	late stage 2			5.1 [2.1–5.9]	0.8149
	stage 1			0.0 [0.0–0.0]	
**INFγ**	early stage 2			0.0 [0.0–7.5]	
	late stage 2			4.3 [0.0–10.4]	0.1367

1 Interquartile range.

2 P values are given for the three class comparisons (stage1, early stage 2, and late stage 2), p-values<0.05 are in bold.

### Plasma cytokine profiles in SERO TL+, HAT patients and endemic controls

**Figure 1:**
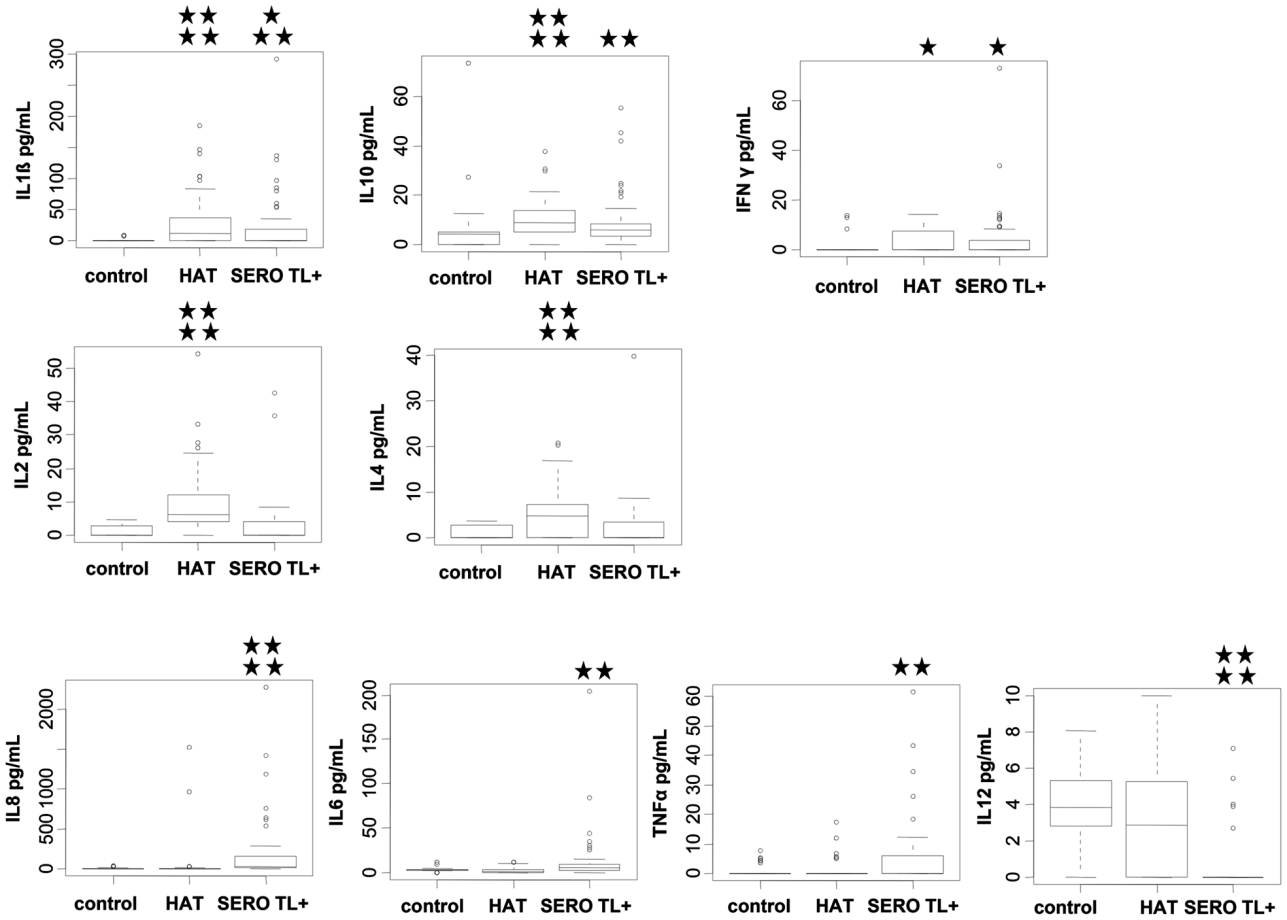
Plasmatic cytokine profiles in HAT patients, SERO TL+ subjects, and endemic controls. Box plots of cytokine concentration are presented. Boxes represent the medians and interquartile ranges and whiskers represent 10^th^ and 90^th^ percentiles. Asterisks indicate significant differences over the control group: ****, p<0.0001; ***, p<0.001; **, p<0.01; *, p<0.05 (nonparametric Wilcoxon signed-rank test). Only cytokines with significant differences are shown. Group effectives: control (n = 40); HAT (n = 52); SERO TL+ (n = 60).

With the exception of IL5, significant differences were observed between HAT patients, SERO TL+ and endemic controls for all other cytokines measured in plasma (Figure 1). The lowest plasma levels of all cytokines except for IL12 were observed in endemic controls. IL1β, IL10, and INFγ cytokine levels were significantly higher in both HAT patients (p<0.0001, p<0.0001, p = 0.01, respectively) and SERO TL+ individuals (p = 0.007, p = 0.004, p = 0.03 respectively) than in endemic controls. IL2 (P<0.0001) and IL4 (p<0.0001) were significantly higher in HAT patients specifically, whereas IL8 (P<0.0001), IL6 (p = 0.001), and TNFα (p = 0.005) were significantly higher in SERO TL+ individuals only. SERO TL+ individuals were also characterized by very low levels of IL12 (p<0.0001) as compared to controls and patients.

**Figure 2:**
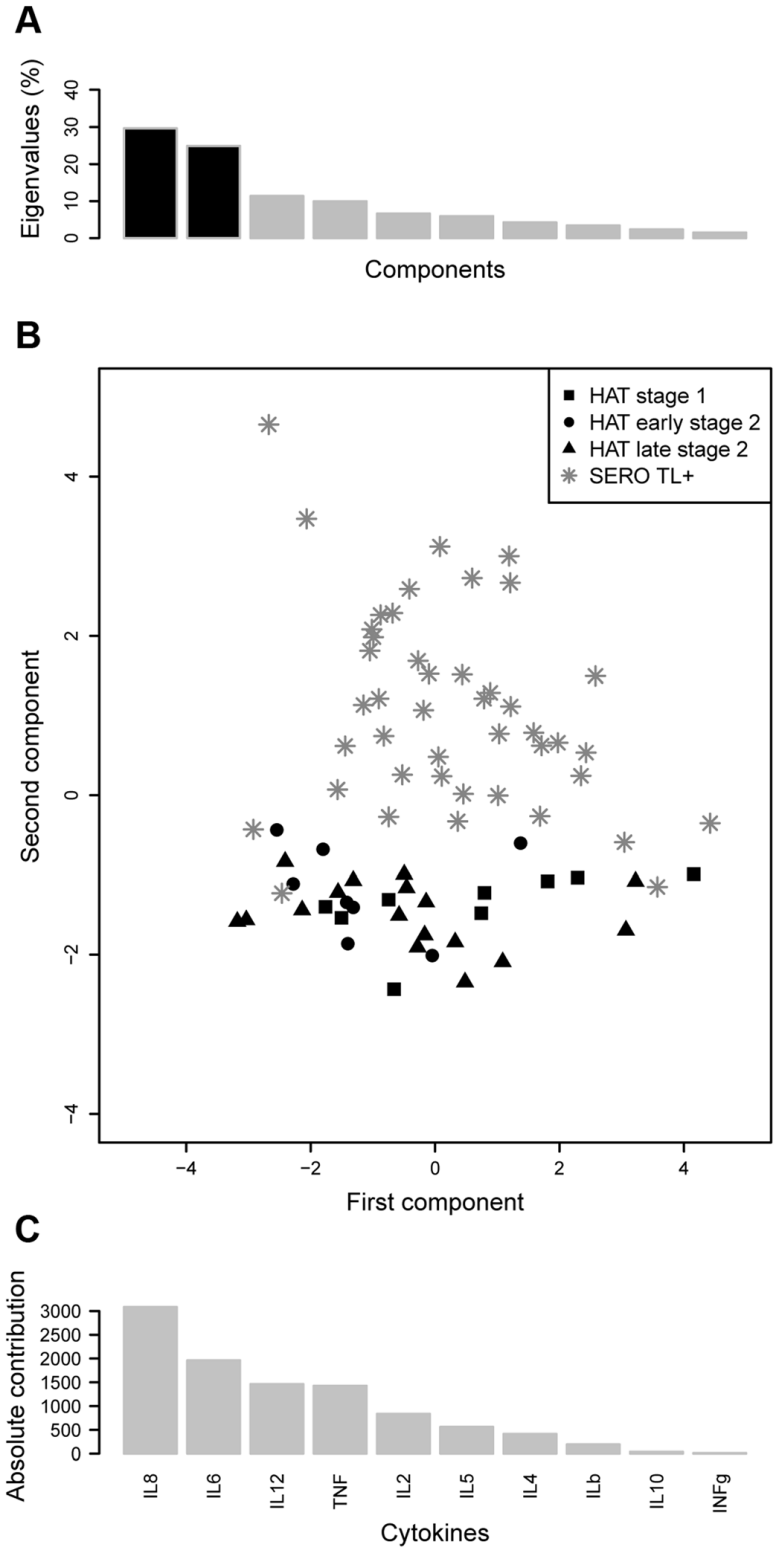
Principal component analysis of cytokine responses in HAT patients and SERO TL+. A. Histogram of eigenvalues. The eigenvalues, which corresponds to the amount of variance accounted by each component, are presented for the first ten components. The two first components, which explain more than 50% of the total variance, are in black. B. First factorial plan (x-axis: first component, y-axis: second component). The plan composed by the two first axis or dimensions is represented. It corresponds to an approximation of the cloud of points by a 2-dimensional space. C. Absolute contribution of each cytokine to the variance of the second component.

In order to get an overview of the cytokine response in SERO TL+ and HAT patients and to explore similarities between individuals or groups of individuals for all the cytokines in a single analysis, we performed a Normed PCA including all 10 cytokines. As shown in Figure 2A the two first components resume more than 50% of the total variance of the data set. A representation of the first factorial plan is given in Figure 2B. None of the recorded covariates (HAT status, disease stage in HAT patients, age, gender, or disease focus) appeared to be correlated with the individuals coordinates on the x-axis. This part of the variance in cytokine levels is likely explained by the occurrence of other pathologies or diseases such as malaria, highly prevalent in the area, but that were not recorded. Nevertheless the second component, which accounted for 24.8% of variation (y-axis), resulted in the separation of study subjects according to a HAT/SERO TL+ gradient. The main cytokines contributing to the variance of the second component were IL8, IL6, IL12, and TNFα, indicating that SERO TL+ are mainly characterized by an inflammatory response, which is not present in HAT patients (Figure 2C).

### Association of cytokine levels with the subsequent evolution of infection in SERO TL+ subjects

**Figure 3:**
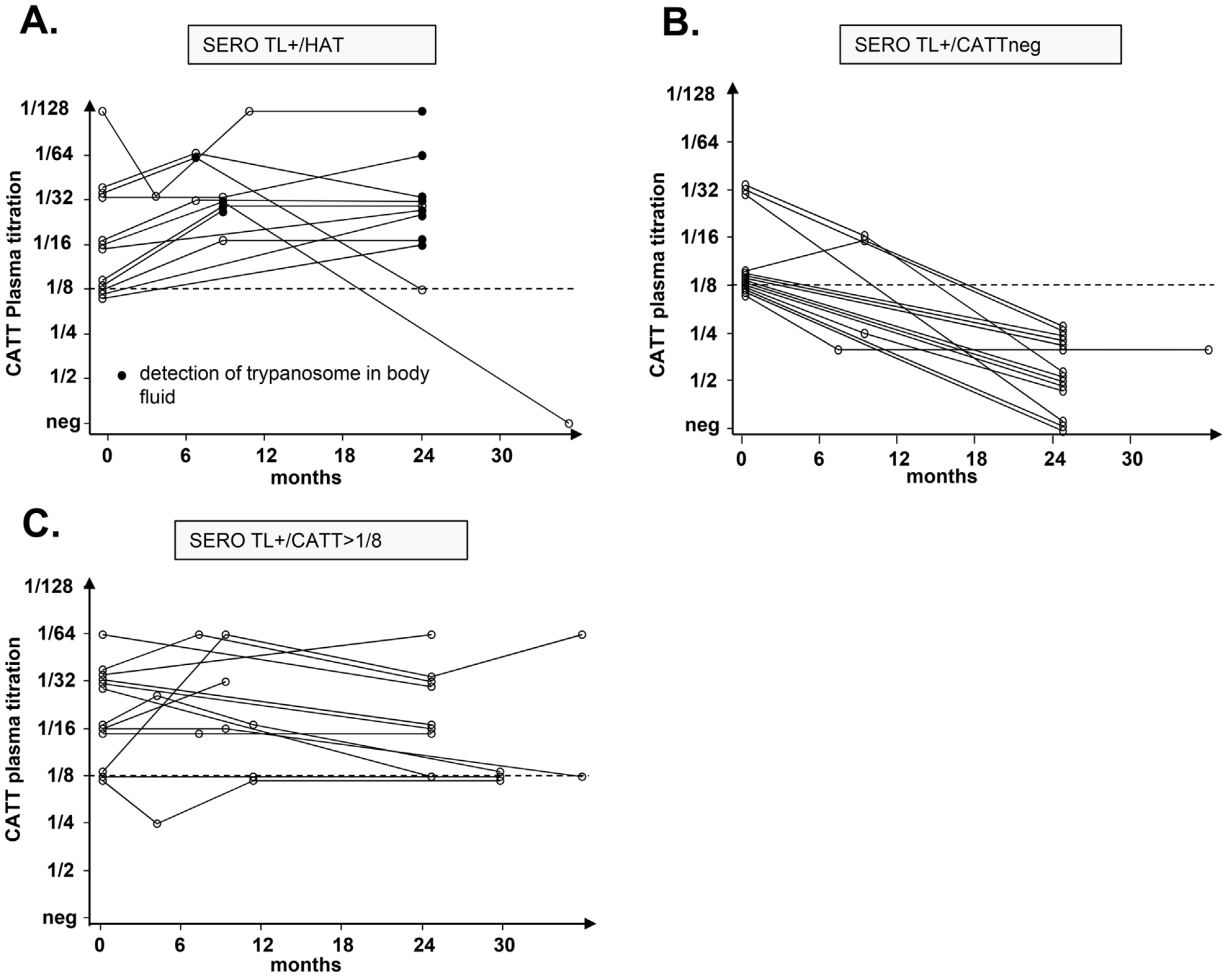
Evolution of the serological and parasitological status in SERO TL+ subjects. The progression of CATT end titers over time is presented for individuals who were confirmed as HAT patients during their follow-up (A.), plain circles indicate that trypanosomes were detected by microscopy in blood or cervical lymph nodes; individuals with decreasing CATT responses (B.) and individuals who maintained high serological responses to the CATT during the follow-up period (C.).

In order to evaluate the prognostic value of the cytokine levels determined at study inclusion on the subsequent evolution of the parasitological and serological status in SERO TL+ individuals, longitudinal follow-up were initiated. According to the results of parasitological and serological tests performed during the follow-up visits we could classify SERO TL+ into three distinct groups (Figure 3). The first group (SERO TL+/HAT) comprised 12 individuals in whom the parasite was detected in body fluids during follow-up. At study inclusion these individuals were presumably in the early stage of the infection process but trypanosomes were not detected at that time. The second group, (SERO TL+/CATTneg) comprised 15 individuals in whom trypanosomes were never detected but who displayed decreasing CATT responses (with end titer becoming <1/8). Similarly decreasing CATT responses were also observed in treated HAT patients in Guinea [19]; they were also observed in confirmed HAT patients refusing treatment in Côte d'Ivoire and in whom parasitological testing became subsequently negative [4], suggesting that these SERO TL+ subjects were engaged in a process of self-cure. The third group (SERO TL+/CATT≥1/8) was composed of 13 individuals who maintained elevated CATT responses throughout the follow-up period and who can be considered as asymptomatic carriers of parasite with parasitemia below the detection limit of parasitological tests.

**Figure 4:**
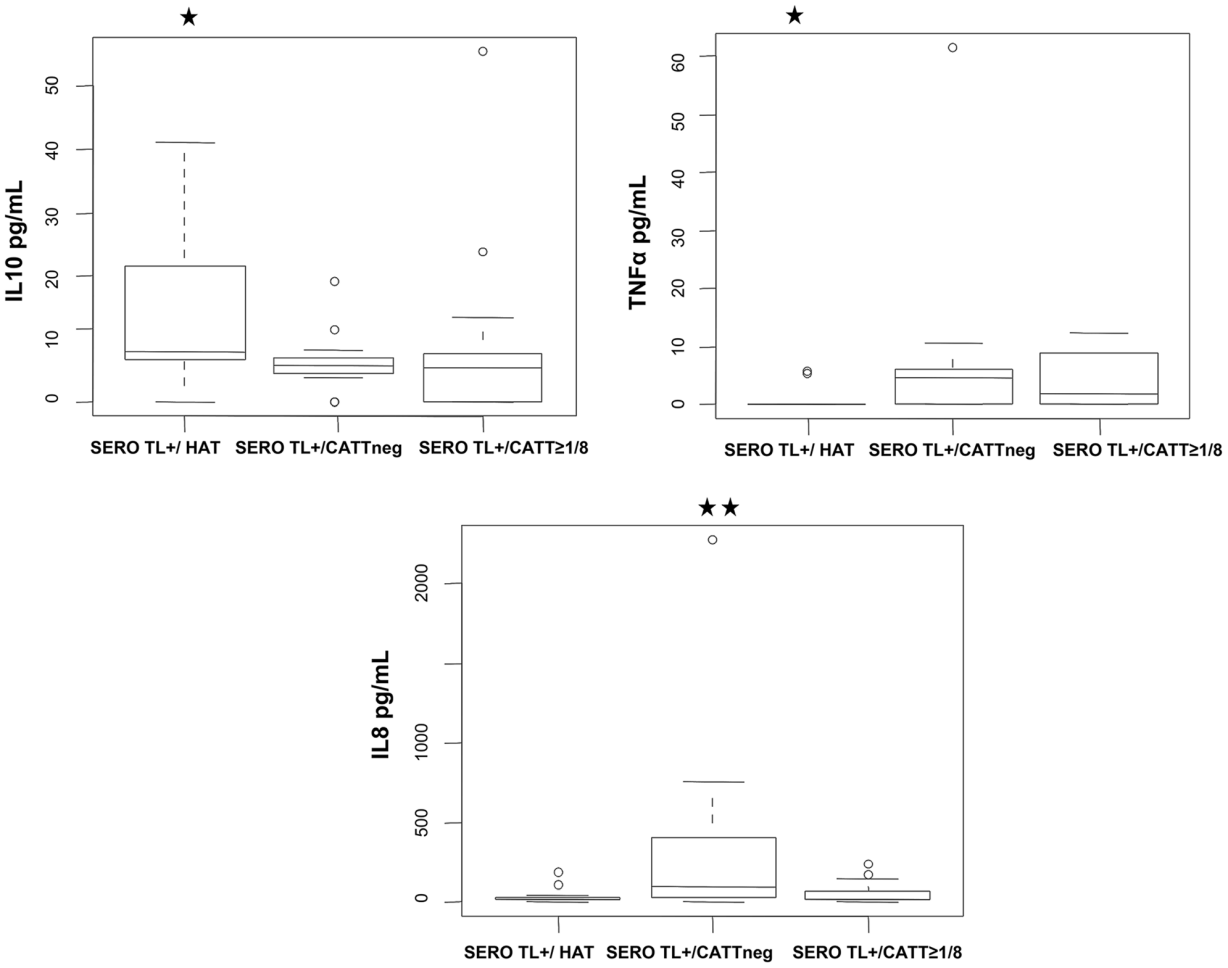
Box-plots of IL10, TNFα, and IL8 concentrations measured in the plasma of SERO TL+ subjects at study inclusion according to follow-up results. SERO TL+/HAT: individuals confirmed as HAT patients (n = 12); SERO TL+/CATTneg individuals with decreasing CATT responses with end titers becoming <1/8 (n = 15); SERO TL+/CATT≥1/8: individuals maintaining elevated CATT responses with end titers ≥1/8 (n = 13). Significant differences in cytokine levels are indicated (**: 0.01<p<0.001; *: 0.05<p<0.01) for a group in comparison with the two others (nonparametric Wilcoxon signed-rank test).

Univariate and multivariate analysis of cytokine levels in these SERO TL+ showed that those individuals with the highest IL10 levels (p = 0.003, OR = 13.09 [2.19–124.29]) and undetectable TNFα (p = 0.009, OR = 10.49 [1.72–101.12]) had a markedly increased risk of developing HAT (Table 3, model I; Figure 4). In contrast, the highest levels of IL8 (Table 3, model II; Figure 4) were significantly associated with the group of SERO TL+ in whom decreasing antibody responses were observed (p = 0.006, OR = 8.32 [1.79–53.44]). We did not observe any significant association of cytokine levels with the maintenance of high CATT responses (Table 3, model III).

**Table 3:** Prognostic value of cytokine levels in SERO TL+.

	covariates with univariate p-value<0.1^1^	covariates included in the model	threshold^2^	p-value	OR^3^	CI^4^
**Model I**						
**SERO TL^+^/HAT**	IL-8 (0.056); IL-10 (0.017); TNFα (0.048);	**IL-10**	**>6 pg/ml**	**0.0034**	**13.09**	**2.19–124.29**
G1 versus G2 and G3	IFNγ (0.098); Age^5^ (0.06)	**TNFα**	**<threshold detection**	**0.009**	**10.49**	**1.72–101.12**
**Model II**						
**SERO TL+/CATTneg**	IL-8 (0.009); IL-6 (0.095); Age (0.066)	**IL-8**	**>30 pg/ml**	**0.0056**	**8.32**	**1.79–53.44**
G2 versus G1 and G3		**Age**	**-**	**0.048**	-	-
**Model III**						
**SERO TL+/CATT≥1/8**	none	none	-	-	-	-
G3 versus G1 and G2						

The prognostic value of cytokine levels measured at inclusion was evaluated by stepwise multivariate logistic regression analysis. For this, SERO TL+ individuals were spited into three categories according to follow-up results: those who were confirmed as HAT patients (SERO TL+/HAT; n = 12; G1), those who presented decreasing CATT responses (SERO TL+/CATTneg; n = 15; G2) and those who maintained high CATT responses and remained negative in parasitology (SERO TL+/CATT≥1/8; n = 13; G3). Three models were then constructed independently by a stepwise procedure comparing SERO TL+/HAT (model I), SERO TL+/CATTneg (model II) and SERO TL+/CATT≥1/8 (model III) to the two other groups.

1 The covariate univariate p-value before the stepwise procedure is shown in between brackets.

2 Cytokine levels were assigned to two class of equal size using the cytokine median value as the threshold.

3 Odds ratio.

4 Confidence interval.

## Discussion

Whereas the concept of trypanotolerance has long been widely accepted and studied in depth in cattle [20] and mice [21], leading to major advances in our understanding of the genetic and immunological mechanisms at play in *T. congolense* and *T. b. brucei* infections [22], [23], knowledge of how *T. b. gambiense* interacts with its hosts lags far behind. One of the reasons is that relevant animal models are not available. In most cases, immunosuppressed animals are required to infect mice with *T. b. gambiense* field isolates; alternatively, old laboratory-adapted strains can be used but may not be very representative of “wild” parasites [24]. A second reason is that immunological studies in human have focused on HAT patients and endemic uninfected controls, thus exploring only part of the spectrum of immune responses, i.e. the one at play in susceptible individuals. In the present study we provide the first insights into the immune response in human hosts (SERO TL+) who are apparently able to control *T. b. gambiense* infection and we have evaluated the prognostic value of cytokine levels on the subsequent evolution of the serological and parasitological status in these subjects.

### Cytokine profile and disease severity in HAT patients

Late-stage HAT develops when trypanosomes cross the blood-brain barrier, inducing a neuroinflammatory process associated with leukocyte infiltration into the central nervous system [25]. As previously observed, elevated levels of cytokines with both inflammatory (IL8 and IL6) and counterinflammatory (IL10) properties were found in the CSF of late-stage patients [12], [13], [26], [27]. These results are in line with the proposal of using these cytokines or other molecules intervening in the neuroinflammatory process [28]–[30] as late-stage diagnostic tools.

In this study we failed to show any significant association between plasma cytokine levels and the different disease stages although trends were observed for IL1β and INFγ to be slightly increased in late stage patients. However we had to rely on limited numbers of subjects for this analysis and this may have precluded finding evidence of small differences in cytokine levels according to the disease stage. Noteworthy, weak association of plasma cytokine levels with disease severity were also observed in other *T. b. gambiense* endemic areas from central Africa [12], [13] although in these studies IL8 levels were slightly higher in early-stage patients. This is in contrast to *T. b. rhodesiense* infections, in which plasma concentrations of TNFα and INFγ were clearly shown to be correlated with disease severity [31], [32]. This may be related to the fact that *T. b. rhodesiense* infections are known to be acute, progressing to late-stage disease in several months, which is in clear contrast to the chronic nature of *T. b. gambiense*.

Nevertheless, important differences in plasma cytokine levels were observed in HAT patients as compared to endemic, uninfected controls, with highly significant differences (p<0.0001) for IL1β, IL2, IL4, and IL10 and to a lesser extent for INFγ (p = 0.01). These results suggest that, in HAT patients, T-cell responses are activated involving both Th1 and Th2 subsets. Although the various animal model systems used have provided conflicting evidence regarding the immunological factors that influence the magnitude of resistance to African trypanosomes, the overall picture in mice is that the host response requires the contribution of both VSG-specific B- and T-cell responses and a proper activation of the macrophage/monocyte phagocyte system to control infection [33]. Type-1 cytokine responses (INFγ, TNFα), leading to macrophage activation to produce trypanotoxic NO [34], are observed during the early stage of infection in both susceptible and resistant mice. However, in resistant mice, the cytokine profile switches to a type-2 response (IL4, IL10) during the late/chronic stages of infection, presumably restricting prolonged and exaggerated inflammatory responses [35]. One has to note that the term “resistant” in mouse models is often exaggerated as these mice are characterized more by chronic infection and delayed mortality, which seems to parallel the infectious course in gambiense HAT. In contrast, early mortality in highly susceptible mice is caused by an excessive activation of the macrophage system, associated with an excessive production of INFγ and a systemic inflammatory syndrome [36], a picture that appears more closely related to human *T. b. rhodesiense* infections.

### Contrasted cytokine profiles are associated with the diversity of infection outcomes

In SERO TL+ individuals the cytokine profile was clearly different from that in HAT patients, although some features were shared such as the induction of IL1β, IL10, and INFγ, although to lower levels than in HAT patients. As shown by the lower levels of IL2 and IL4 and the almost absence of IL12, a cytokine that plays a pivotal role in driving Th1-mediated cellular immunity, T-cell responses appeared to be less activated in SERO TL+. In contrast, SERO TL+ subjects were characterized by a marked inflammatory response with elevated levels of IL8, IL6, and TNFα. In these subjects, this inflammatory process appears to be Th1 independent and thus more likely results from the innate activation of the immune system, possibly through parasite-derived, macrophage-activating molecules interacting with pattern recognition receptors [37], [38].

As previously reported in Guinea [7], follow-up of SERO TL+ individuals over a 2-years period showed that this group is heterogeneous. Interestingly, SERO TL+ with the highest IL10 levels and none detectable TNFα at study inclusion had a markedly increased risk of being confirmed by microscopy as HAT patients during their follow-up (OR = 13.09 [2.19–124.29] and OR = 10.49 [1.72–101.12] respectively). Increased IL10 and low TNFα levels were also observed in HAT patients confirming their association with disease susceptibility. Interestingly a line of evidences indicates that the production of TNFα is involved in the control of parasite growth but also in the development of pathogenesis in experimental trypanosomiasis [39]. The mechanisms by which TNFα interacts with trypanosomes (via direct versus indirect actions) are still controversial and differ in the various experimental models [40], [41]. In the light of our results, one can note that the production of TNFα was shown to be induced by *T. b. gambiense* in human macrophages [42] and that IL10 is well known for its macrophage deactivation properties. Finally, an important finding of this study was that high levels of IL8 (>30 pg/ml, OR = 8.32, p = 0.006) were associated with the group of SERO TL+ becoming negative in serology, suggesting that this cytokine is a marker of the host immune response able to eliminate infection. IL8 is a major cytokine involved in innate immune responses. Its main function is to be a chemoattractant for neutrophils, suggesting that these cells play an important role in resistance to infection. In contrast to mice, neutrophils represent 50–70% of leukocytes in humans; they are essential effectors of innate immunity notably through the production of antimicrobial peptides [43], among which the cathelicidins were shown to be typanotoxic [44].

**Figure 5:**
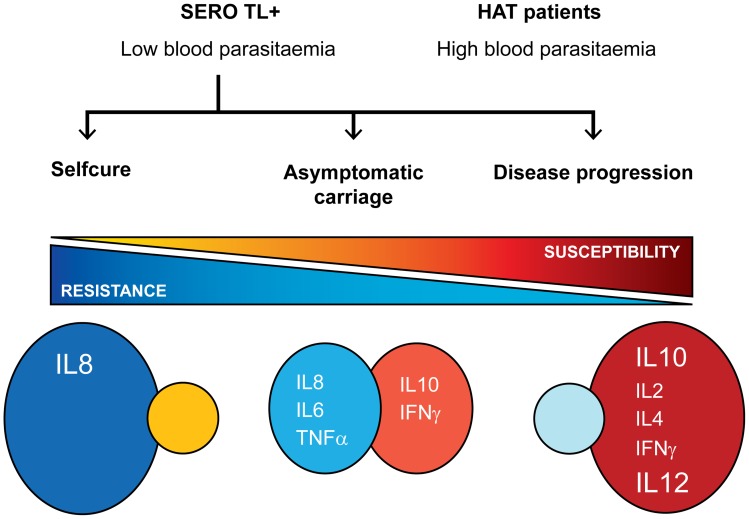
Cytokine profiles and infection outcomes in gambiense HAT.

Whereas the analysis of several cytokines only provides a limited view of the host immune response to infection and precludes drawing firm conclusion on the precise mechanisms controlling parasite growth and or pathogenesis in human, for the first time this study shows that contrasted cytokine profiles (summarized in Figure 5) are associated with the diversity of infection outcomes observed in *T. b. gambiense* endemic areas [1]. Unraveling the mechanisms underlying human resistance/susceptibility to *T. b. gambiense* and identifying the key controlling elements will require further studies. Understanding how the human host is naturally able to control and even cure infection is an exciting issue with the potential of identifying novel therapeutic targets. Ongoing blood mRNA profiling and whole genome association studies comparing HAT patients and SERO TL+ individuals [45] will hopingly help unraveling the mechanisms underlying human resistance/susceptibility to *T. b. gambiense*.

### New tools to improve the management of seropositive subjects?

As part of the HAT elimination goal, increasing attention is being paid to asymptomatic carriers. As available treatments are expensive, still toxic, and require long-term hospitalization in specific treatment centers, unconfirmed serological suspects are usually not treated and sent back home by the mobile teams screening the population. Regarding this situation, an important finding of the present study is that markers of disease development (high IL10, low TNFα) or self-cure processes (high IL8) exist in the blood of SERO TL+. These cytokines, or other biomarkers yet to be discovered, could thus be used to develop simple tests that help make the appropriate therapeutic decision for this particular category of subjects. Further studies based on larger samples from different endemic areas will have to be performed to confirm the prognostic value of these cytokines or other markers and define the most appropriate concentration threshold to consider in order to reach optimum specificity.

### Conclusion

To our knowledge this study is the first to show that human individuals able to resist *T. b. gambiense* are characterized by a marked inflammatory cytokine profile, pointing out innate immunity and possibly neutrophils as potential actors involved in the control of infection. Future and ongoing studies using high throughput methods to compare HAT patients and SERO TL+ will provide a more precise picture of the immune response occurring in these individuals and hopingly help identifying the critical molecules or pathway controlling resistance/susceptibility to *T. b. gambiense* HAT. We believe such studies will provide new insights into the identification of novel therapeutic or prophylactic targets and enable the design of new tools to improve the diagnosis and management of parasitologicaly unconfirmed seropositive individuals, an important challenge in the perspective of HAT elimination.

## Supporting Information

Figure S1 **Box-plots of cytokine concentrations measured in the plasma of HAT patients according to the disease stage.** Boxes represent the medians and interquartile ranges and whiskers represent 10^th^ and 90^th^ percentiles. Group effectives: stage 1 (n = 10); early stage 2 (n = 19); late stage (n = 23). No significant differences between groups were detected (Kruskal-Wallis nonparametric one-way analysis of variance).
(TIF)
