# Supplementary figures and images for: Unravelling Human Trypanotolerance: IL8 is Associated with Infection Control whereas IL10 and TNFα Are Associated with Subsequent Disease Development
Source: PLoS Pathog. 2014 Nov 6;10(11):e1004469. doi: 10.1371/journal.ppat.1004469 (PMC4223068; doi:10.1371/journal.ppat.1004469)

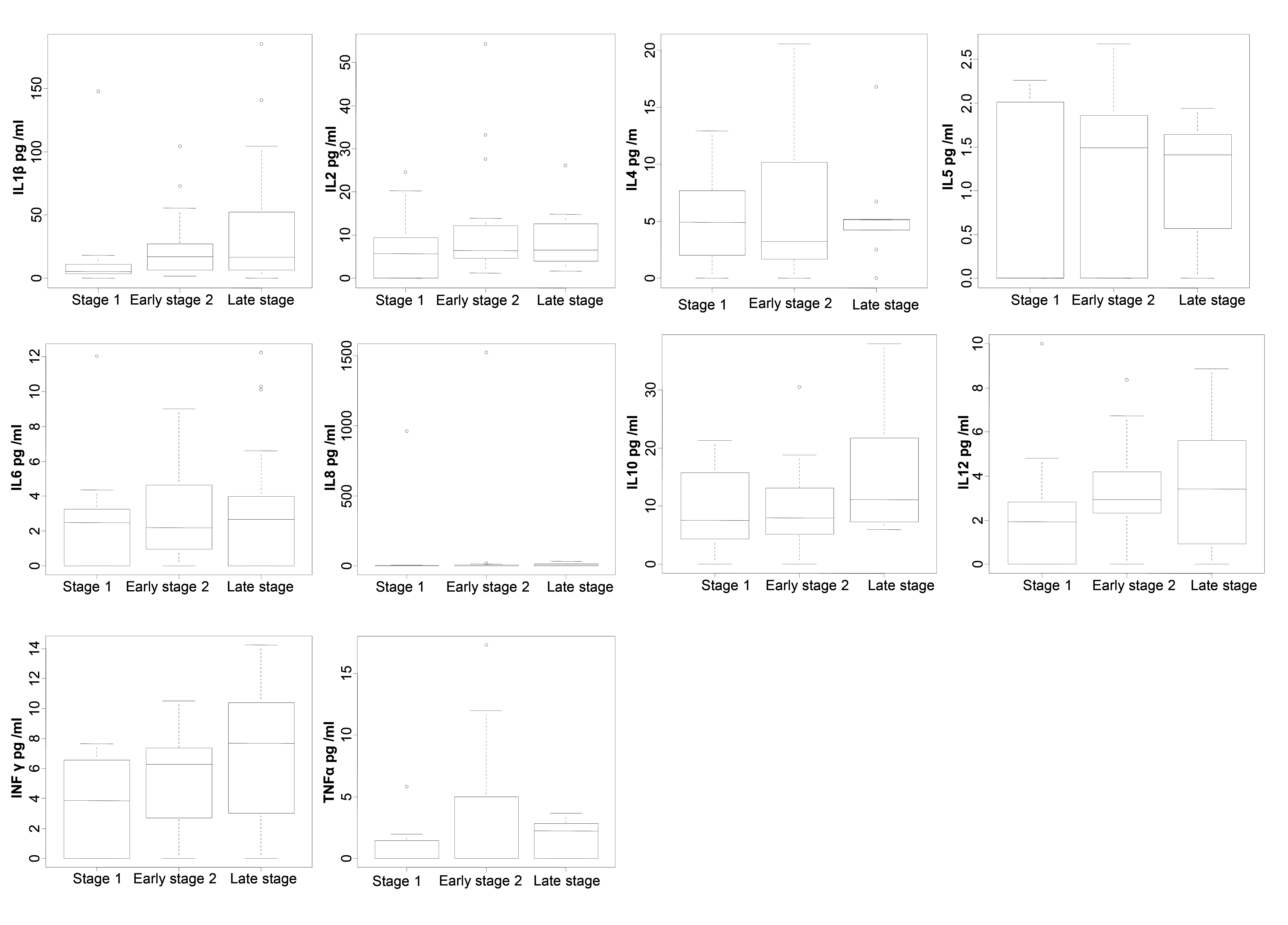

Supplement: Figure S1 — Box-plots of cytokine concentrations measured in the plasma of HAT patients according to the disease stage. Boxes represent the medians and interquartile ranges and whiskers represent 10th and 90th percentiles. Group effectives: stage 1 (n = 10); early stage 2 (n = 19); late stage (n = 23). No significant differences between groups were detected (Kruskal-Wallis nonparametric one-way analysis of variance). (TIF) [file ppat.1004469.s001.tif]
